# Saphenous Vein Vasa Vasorum as a Potential Target for Perivascular Fat-Derived Factors

**DOI:** 10.21470/1678-9741-2020-0031

**Published:** 2020

**Authors:** Andrzej Loesch, Michael Richard Dashwood

**Affiliations:** 1 Centre for Rheumatology and Connective Tissue Diseases, University College London Medical School, Royal Free Campus, London, United Kingdom.; 2 Division of Surgery and Interventional Science, University College London Medical School, Royal Free Campus, London, United Kingdom.

**Keywords:** Saphenous Vein, Adventitia, Perivascular Fat, Vasa Vasorum, Microvessels, Nutrients

## Abstract

Perivascular adipose tissue (PVAT) is a source of factors affecting vasomotor tone with the potential to play a role in the performance of saphenous vein (SV) bypass grafts. As these factors have been described as having constrictor or relaxant effects, they may be considered either beneficial or detrimental. The close proximity of PVAT to the adventitia provides an environment whereby adipose tissue-derived factors may affect the vasa vasorum, a microvascular network providing the vessel wall with oxygen and nutrients. Since medial ischaemia promotes aspects of graft occlusion the involvement of the PVAT/vasa vasorum axis in vein graft patency should be considered.

**Table:** 

Abbreviations, acronyms & symbols				
**A**	**= Adventitia**	** **	**I-M**	**= Intima/media**
**A-M**	**= Adventitial medial**		**Lu**	**= Lumen**
**ADRFs**	**= Adipocyte-derived relaxing factors**		**M**	**= Media**
**BK**	**= Bradykinin**		**NA**	**= Noradrenaline**
**bv**	**= vasa vasorum blood vessel**		**NO**	**= Nitric oxide**
**CABG**	**= Coronary artery bypass grafting**		**NS**	**= No significant**
**CGRP**	**= Calcitonin gene-related peptide**		**NT**	**= No-touch**
**CT**	**= Conventional**		**NTSV**	**= No-touch saphenous vein**
**CTSV**	**= Conventionally harvested saphenous vein**		**PE**	**= L-phenylephrine**
**eNOS**	**= Endothelial nitric oxide synthase**		**PVAT**	**= Perivascular adipose tissue**
**ET-1**	**= Endothelin-1**		**PVT**	**= Perivascular tissue**
**ETA**	**= Endothelin receptor A**		**SNP**	**= Nitroprusside**
**H2S**	**= Hydrogen sulfide**		**SP**	**= Substance P**
**I**	**= Intima**		**SV**	**= Saphenous vein**

Currently, there is much interest on the influence of perivascular adipose tissue (PVAT) on vessel structure and function; in particular, the possible effects of PVAT on the patency of human saphenous vein (SV) used as coronary artery bypass grafts. A recent study published by Yamada et al.^[1]^ provides interesting data related to the preserved vasomotor properties of SV grafts harvested by a no-touch technique (NT) for coronary artery bypass grafting (CABG). There, it has clearly been demonstrated that both the vasoconstrictor and vasorelaxant properties of no-touch saphenous vein (NTSV) grafts were preserved, which the authors attribute to the maintained integrity of the SV graft during NT harvesting. Hence, preservation of the adventitia, media, and intima combined with the histological confirmation of the vessel’s normal architecture support the suggestion that reduced vessel damage is critical for improved graft performance. This is in contrast to conventionally harvested SV (CTSV), where the adventitia is stripped/damaged and the vein distended, resulting in additional medial and intimal damage. We question the reference to Lynch et al.^[2]^, by Yamada et al.^[1]^, when discussing the NT technique, as this study relates to vasomotor responses of small arteries from wild-type and large-conductance Ca^2^+-activated K^+^ channel knockout mice, although it is true that this study investigated the reactivity of vessels in the presence and absence of PVAT. Therefore, this study does bear some similarity with the NT technique. Nonetheless, when studying human NTSV, it is more appropriate to cite the technique pioneered by Souza, who introduced the NTSV harvesting technique for CABG surgery over 20 years ago^[3]^. Apart from these observations, the results described by Yamada et al.^[1]^ are striking, especially their demonstration that PVAT is a source of vasoconstrictor factor(s).

According to Yamada et al.^[1]^, NT SV segments exhibited greater constrictor responses to both potassium chloride and L-phenylephrine (PE) than conventional (CT) segments. Non-endothelial relaxation in response to nitroprusside (SNP) was greater in CT than in NT segments at the low SNP dose (10^−8.0^ mol/L); but at the high SNP dose (10^−5.5^ mol/L), NT relaxation was greater than CT. While the SNP data was inconsistent, the relaxant effects of bradykinin (BK) in segments pre-constricted with PE were significantly greater in CT than in NT, an effect that was nitric oxide (NO)-dependent, based on incubation in the presence of the NO synthase-inhibitor L-NG-nitro arginine methyl ester. These results are somewhat in contrast to data from similar earlier studies. For example, the presence of PVAT in SV preparations has been shown to significantly attenuate the contractile response to noradrenaline (NA), an effect due to both prostaglandin E2 and prostacyclin (Ozen et al.)^[4]^. The study by Momin et al.^[5]^ showed that the adipokine leptin had a relaxant effect on human SV that was neither NO nor endothelial-dependent. Another potential adipocyte-derived factor implicated in SV graft performance is H_2_S, based on a study using a combination of *in vitro* functional immunohistochemistry and electron microscopy studies^[6]^.

The existence of a PVAT-derived, transferable, anti-contractile factor was previously identified in similar organ bath studies 15 years ago using the internal thoracic artery, the gold standard graft for CABG (Gao et al.^[7]^). These, and many other recent publications, lend strong support for the existence of the so-called adipocyte-derived relaxing factors (ADRFs), that were first described in detail in the excellent review by Gollasch and Dubrovska^[8]^. In the subsequent years, numerous reviews on ADRFs have been published^[9-11]^, including those discussing the potential importance of the preservation of PVAT on NTSV grafts^[12-14]^ (Figure 1) and its role in their improved patency when compared to CT grafts at up to a 16-year follow-up (Samano et al.^[15]^). As it is evident from the abovementioned studies, PVAT has been shown to play a role in the reactivity of SV used as grafts in CABG^[13,16]^. However, there may be some debate as to whether these effects are in fact “relaxant” or “anti-contractile”.

**Fig. 1:**
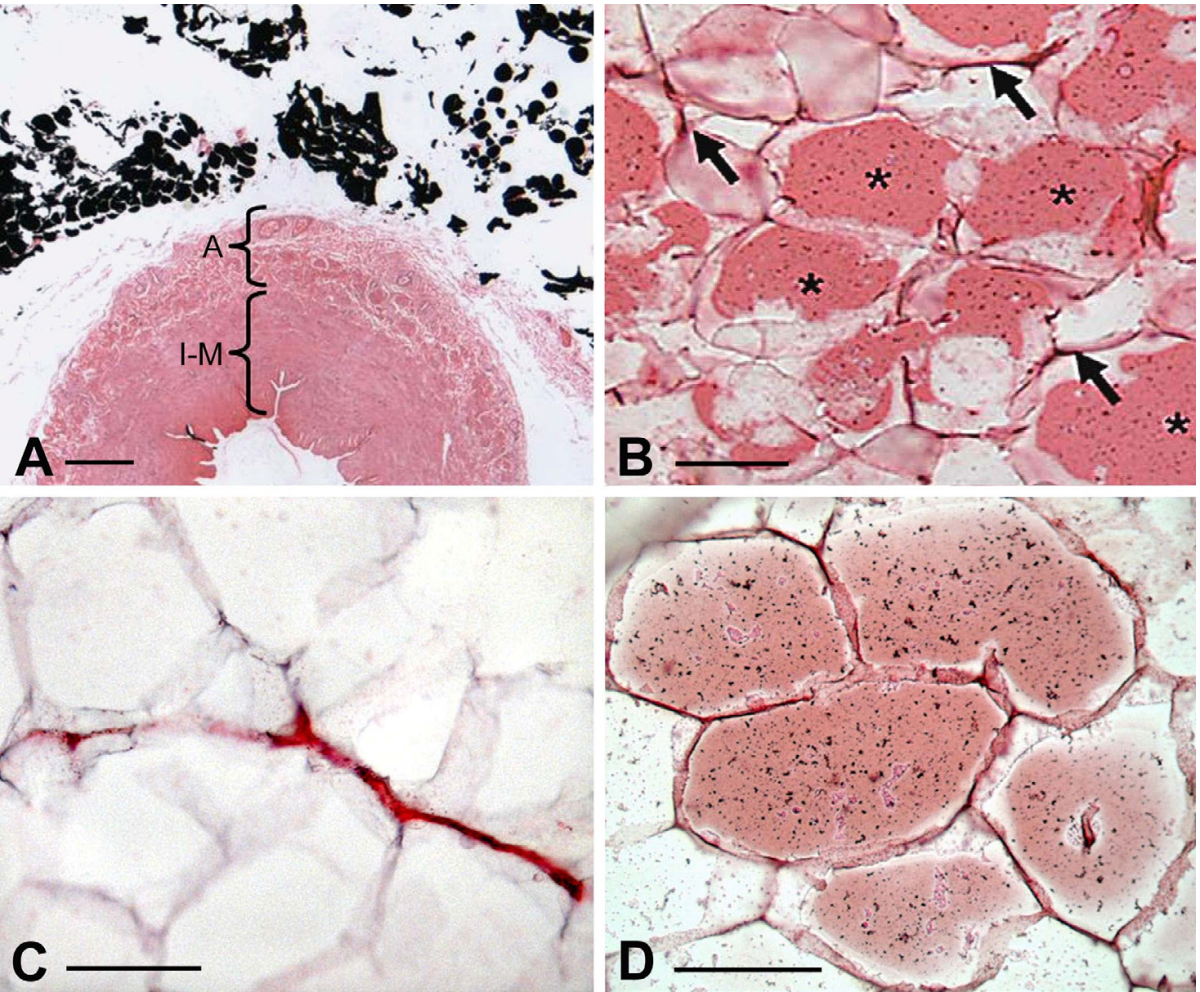
Presence of perivascular adipose tissue (PVAT) in no-touch saphenous vein (SV) harvested for coronary artery bypass grafting. A) Part of a transverse section of SV with PVAT intact (fat stains black using the Marchi technique). A=adventitia; I-M=intima/media. Bar: 0.5 mm. B) Endothelial nitric oxide synthase (eNOS) immunostaining of SV PVAT (*). Arrows indicate eNOS staining of capillaries. Bar: 50 µm. C) Capillaries in PVAT where the endothelium is identified by cluster of differentiation 31. Bar: 50 µm. D) Leptin immunostaining of SV PVAT. Bar: 50 µm. It is acknowledged that images are modified from own work: A and B) Dashwood et al.^[12]^, 2007; C) Fernandez-Alfonso et al.^[13]^, 2016; D) Dashwood et al.^[14]^, 2011.

As the microvascular network provides the SV wall with oxygen and nutrients^[17,18]^, what role might the vasa vasorum and its preservation in SV play in graft patency? Using a myograph, a miniaturised version of the organ bath, the reactivity of vasa vasorum has been studied. The vasa vasorum of porcine and bovine thoracic aorta were mounted in a myograph to study the effect of constrictor and relaxant compounds (Scotland et al.^[19]^). The constrictors used were endothelin-1 (ET-1), NA, angiotensin II, and thromboxane A2-mimetics (U44069 or U46619) and vasodilators; substance P (SP), BK, calcitonin gene-related peptide (CGRP), or isoprenaline were also used. Potent concentration-dependent contraction of vasa vasorum was produced by ET-1, whereas NA was a weak constrictor. SP and BK produced endothelium-dependent relaxation, while CGRP produced endothelium-independent relaxation of ET-1-preconstricted vasa vasorum. An interesting observation from these studies, when comparing isolated vasa vasorum and strips of aorta, was the fact that the vasa vasorum appear to respond to constrictors differently from the large host vessel. The authors concluded that “…various pathophysiological states might significantly alter the reactivity of the vasa vasorum which in turn may lead to underperfusion of the host vessel wall. In this respect endothelin receptor antagonists might have useful effects to preserve or restore patency of the vasa vasorum”^[19]^.

This suggestion seems most credible, particularly in the light of data from studies published 20 years ago. There, adventitial placement of a constrictive collar around the rabbit carotid artery caused neointimal formation, an effect due to macrophage and smooth muscle cell infiltration into the arterial subendothelium, foam cell formation, and the deposition of extracellular lipid. This effect was repeated in a number of subsequent studies with the authors concluding “It is proposed that the changes induced by the collar may be mediated by obstruction of the adventitial vasa vasorum with the creation of a localised ischaemic region”^[20-22]^.

A number of constrictor and dilator factors have been identified that are associated with vasa vasorum of SV used in CABG, including ET-1^[23]^, 5-hydroxytryptamine^[24]^, leptin, and endothelial nitric oxide synthase (eNOS)^[12,14]^. However, there are marked differences when comparing the density of vasa vasorum of SV harvested by CT *vs*. NTSV grafts, as well as between venous and arterial grafts (Dreifaldt et al.^[25]^). It may be reasonable to assume that stimulation of these receptors is associated with both the constrictor and dilator effects observed in *in vitro* studies^[19]^. Furthermore, drug-induced constriction of the vasa vasorum would be expected to produce the same effect as occlusion of the vasa vasorum by placement of an adventitial collar^[20-22]^. This raises the question as to whether targeting the vasa vasorum with the relevant drugs might offer therapeutic potential in improving SV graft patency. For example, the endothelin receptor A (ETA) receptor antagonist, BSF 302146, has been shown to improve graft patency in a porcine SV to carotid artery interposition model (Wan et al.^[26]^). There, neointimal hyperplasia was dramatically reduced in SV grafts of pigs receiving the ETA receptor antagonist where a high density of both ET-1 and its receptors was identified, not just within regions of neointimal thickening, but also with proliferating vasa vasorum^[26]^. Might such results be possible in patients undergoing CABG receiving SV grafts? Of course, drug targeting would be an issue, although various studies describe beneficial effects using local delivery by gene transfer^[27]^ or drug eluting stents^[28]^. The question is: do PVAT-derived factors affect vasa vasorum tone and, therefore, blood flow through the media? According to some, PVAT factors are constrictors^[1,16,29]^, and this is “bad”. Are there compounds/drugs that might counteract this (*e.g.*, “fish oils”) and improve blood supply in pathological conditions?

While the study by Yamada et al.^[1]^ shows that PVAT possesses constrictor activity, others have shown it to have a relaxant or anti-contractile action^[29-32]^. This is particularly pertinent when considering the improved performance of SVs harvested by the NT technique, where the vein is harvested complete, with its surrounding cushion of PVAT intact (Souza^[3]^). This cushion of perivascular fat exhibits positive immunostaining for eNOS, the enzyme responsible for producing NO, a potent vasodilator with anti-proliferation and anti-thrombotic properties^[12]^. Also, eNOS protein expression and NO production have been demonstrated in tissue extracts of PVAT from NTSVs^[12]^. The conflicting results regarding whether PVAT possesses constrictor or dilator activity is not uncommon and is discussed in detail in the review, “Role of PVAT in coronary atherosclerosis and vein graft patency: friend or foe?” by Fernández-Alfonso et al.^[16]^. There a ‘bidirectional paradigm’ is outlined whereby signals initiated from the lumen may affect the PVAT resulting in either beneficial or deleterious consequences. More recently, an inside out/outside in communication of the vasa vasorum has been proposed to play a role in the patency rate of the SV when used as a bypass graft in CAB^G[33)^. This suggestion is supported by the observation of potential luminal termination of vasa vasorum in the SV^[25,34]^, observations in support of a statement appearing 70 years ago in an early edition of Ham’s Histology textbook^(35].^ Experimentally, using close fitting external collars or removal of the adventitia, interruption of blood supply to the media plays an important role in neointima and atheroma formation^[20-22]^. When performing CT harvesting, where pedicle including PVAT is removed, the adventitial vasa vasorum is severed^[36,37]^ (Figure 2), and the medial vasa vasorum exhibit plugging of red blood cells^[38]^, both features that will also reduce medial blood supply, resulting in ischaemia and neointimal thickening^[21,22,39]^. In respect to SV coronary grafts, however, most of the papers discussing the mechanisms of neointimal thickening/hyperplasia do not relate this phenomenon to poor blood supply to the vein wall, focussing mostly on damage to the endothelium and vascular smooth muscle proliferation, hence overlooking the role and contribution of the vasa vasorum^[40-43]^.

**Fig. 2:**
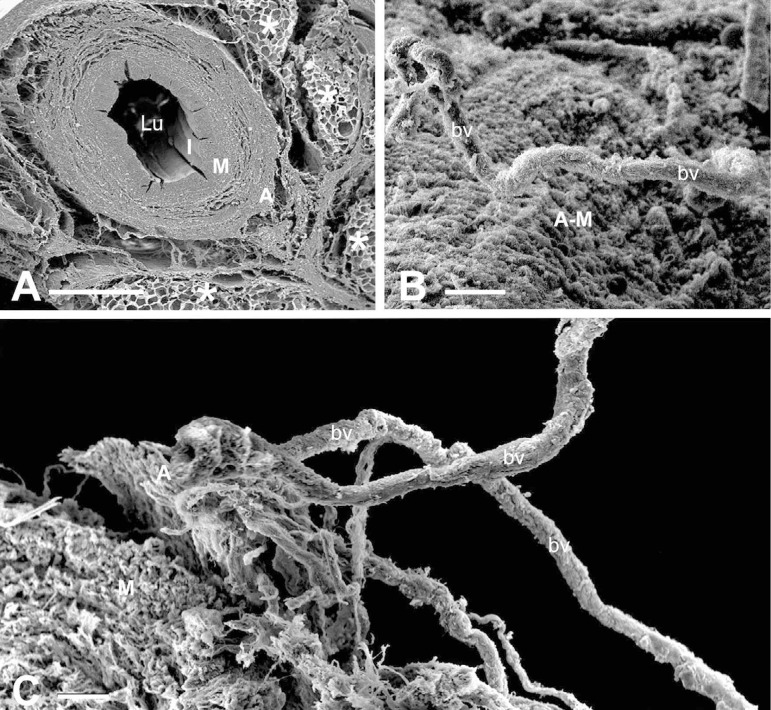
Scanning electron microscopy examples of no-touch saphenous vein (NTSV) and conventionally harvested saphenous vein (CTSV) for CABG. A) In a transverse section, note a general architecture of NTSV showing lumen (Lu), intima (I), media (M), adventitia (A), and the intact cushion of surrounding tissue, much of which is perivascular adipose tissue (*). Bar: 1 mm. B) An en face view of an adventitial-medial (A-M) border of CTSV showing an exposed vasa vasorum blood vessel (bv). Bar: 50 µm. C) In CTSV, note that the vasa vasorum vessels together with a remnant of A are almost detached from M and exposed to the external environment. Bar: 50 µm. It is acknowledged that images are modified from own work: A) Dashwood et al.^[36]^, 2004; B and C) Vasilakis et al.^[37]^, 2004.

There is the possibility that removal of the PVAT in CT harvesting reduces, or abolishes, the beneficial effect of ADRFs on the vasa vasorum and that this plays an important role in the inferior patency of CTST *vs*. NTSV grafts. There is also the possibility that bidirectional transport of PVAT-derived factors through the vasa vasorum is affected. Such issues may be difficult to address when using organ bath studies as described by Yamada et al.^[1]^ and others^[4,6,7]^, but they may perhaps be elucidated using the myograph preparation, although this is technically more challenging. In fact, using this technique, Vestergaard et al.^[44]^ showed a tendency toward improved vasorelaxation of NTSV compared to CTSV (Figure 3), but this improvement was just below statistical significance. However, as some NTSVs examined were manually distended (no details provided) and some were not distended, it is not clear if both distended and non-distended NTSVs were analysed together as one group. Until this is clarified, therefore, the true comparison of vasoreactivity of CTSV with “real” NTSV (not stripped and not distended) is unknown.

**Fig. 3:**
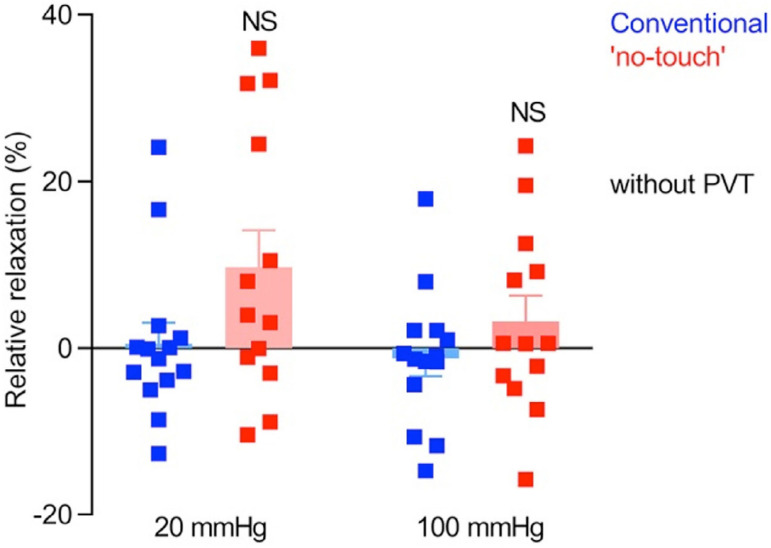
Diagram shows variability of endothelium-dependent relaxation of human saphenous vein harvested by conventional (blue) and “no-touch” (red) techniques. Although there is no significant (NS) difference between grafts harvested by either of techniques, there is a tendency to increased relaxation properties of ‘no-touch’ grafts. The vein grafts (n = 13-14) were stretched to an internal diameter corresponding to a transmural pressure of 20 or 100 mmHg, pre-contracted with 10 µM serotonin, and exposed to 10 µM methacholine. It is acknowledged that this diagram is from the Open Access article Vestergaard et al.^[44]^, 2018. PVT=perivascular tissue.

Following the original introduction of the NTSV harvesting technique by Souza^[3]^, there have been various attempts to further develop and/or modify this technique of harvesting the SV for CABG. In fact, these attempts are in recognition of the superiority of NTSV grafts over CTSV harvesting. The most recent example of such appreciation of NTSV harvesting is the description of a minimally invasive endoscope approach using bipolar electrothermy, where the SV graft was harvested with a pedicle of surrounding tissue (including PVAT), and where the intima, media, adventitia and vasa vasorum appeared histologically normal. This indicates that there was no vascular damage inflicted during harvesting, suggesting good prognosis for graft patency (Hayashi et al.^[45]^). The important clinical advantage - the improved patency - of NT *vs*. CT SV grafts for CABG surgery is clear^[15]^. The fact remains that SV is still the most commonly used vessel as a coronary graft in many countries, for example, a recent study in Brazil showed that SV accounts for around 84%, as the second conduit of choice for CABG^[46,47]^.

## CONCLUSION

Perivascular fat surrounding the SV has been shown to produce either constrictor or relaxant effects *in vitro*, effects that may play a role in graft performance in patients undergoing CABG. Fat-derived factors have been suggested to play both detrimental and beneficial effects on vein graft performance. While such factors may have a direct action on the vascular smooth muscle cells of the media, there is also the possibility that they act on the vasa vasorum, the microvascular network supplying the media with oxygen and nutrients. Reduced medial blood flow promotes vascular smooth muscle cell proliferation, neointimal hyperplasia, and atheroma formation, leading to eventual graft occlusion. When examining the role of perivascular fat on SV graft patency the potential involvement of the vasa vasorum should be considered.

**Table:** 

Authors' roles & responsibilities	
AL	Substantial contributions to the conception or design of the work; drafting the work or revising it critically for important intellectual content; agreement to be accountable for all aspects of the work in ensuring that questions related to the accuracy or integrity of any part of the work are appropriately investigated and resolved; ﬁnal approval of the version to be published
MRD	Substantial contributions to the conception or design of the work; drafting the work or revising it critically for important intellectual content; agreement to be accountable for all aspects of the work in ensuring that questions related to the accuracy or integrity of any part of the work are appropriately investigated and resolved; ﬁnal approval of the version to be published
